# The Herpes Virus Fc Receptor gE-gI Mediates Antibody Bipolar Bridging to Clear Viral Antigens from the Cell Surface

**DOI:** 10.1371/journal.ppat.1003961

**Published:** 2014-03-06

**Authors:** Blaise Ndjamen, Alexander H. Farley, Terri Lee, Scott E. Fraser, Pamela J. Bjorkman

**Affiliations:** 1 Division of Biology and Biochemical Engineering, California Institute of Technology, Pasadena, California, United States of America; 2 Howard Hughes Medical Institute, California Institute of Technology, Pasadena, California, United States of America; University of Glasgow, United Kingdom

## Abstract

The Herpes Simplex Virus 1 (HSV-1) glycoprotein gE-gI is a transmembrane Fc receptor found on the surface of infected cells and virions that binds human immunoglobulin G (hIgG). gE-gI can also participate in antibody bipolar bridging (ABB), a process by which the antigen-binding fragments (Fabs) of the IgG bind a viral antigen while the Fc binds to gE-gI. IgG Fc binds gE-gI at basic, but not acidic, pH, suggesting that IgG bound at extracellular pH by cell surface gE-gI would dissociate and be degraded in acidic endosomes/lysosomes if endocytosed. The fate of viral antigens associated with gE-gI–bound IgG had been unknown: they could remain at the cell surface or be endocytosed with IgG. Here, we developed an in vitro model system for ABB and investigated the trafficking of ABB complexes using 4-D confocal fluorescence imaging of ABB complexes with transferrin or epidermal growth factor, well-characterized intracellular trafficking markers. Our data showed that cells expressing gE-gI and the viral antigen HSV-1 gD endocytosed anti-gD IgG and gD in a gE-gI–dependent process, resulting in lysosomal localization. These results suggest that gE-gI can mediate clearance of infected cell surfaces of anti-viral host IgG and viral antigens to evade IgG-mediated responses, representing a general mechanism for viral Fc receptors in immune evasion and viral pathogenesis.

## Author Summary

Herpes Simplex Virus 1 (HSV-1) infects 40–80% of adults worldwide. HSV-1 initiates infection at mucosal surfaces and spreads along sensory neurons to establish a life-long latent infection that can lead to neurological diseases. Humans usually develop IgG antibodies that specifically recognize pathogens via fragment antigen binding (Fab) variable regions. HSV-1 can avoid the protective effects of antibodies by producing gE-gI, a receptor that binds to the constant portion of IgGs (Fc), thereby tethering the antibody in a position where it cannot trigger downstream immune functions. A gE-gI–bound IgG can participate in antibody bipolar bridging (ABB) such that the Fabs bind a viral antigen and the Fc binds gE-gI. The fate of ABB complexes had been unknown. We used live cell fluorescent imaging to follow ABB complexes during their formation and transport within a cell. We demonstrated that ABB assemblies were internalized into acidic intracellular compartments, where gE-gI dissociated from IgG–viral antigen complexes and the IgG and antigen were targeted for degradation within lysosomes. These results suggest that gE-gI mediates clearance of infected cell surfaces of both anti-viral IgGs and viral antigens, a general mechanism to facilitate latent infection by evading IgG-mediated responses.

## Introduction

Herpes Simplex Virus (HSV), Varicella-Zoster Virus (VZV), and Pseudorabies Virus (PrV) are members of the alpha herpes virus family, which are characterized by a relatively short replicative cycle in epithelial tissues and egression to and latent infection of the sensory neurons [1]–[5]. Alpha herpes viruses have evolved many strategies to evade the host immune system. For example, antibodies do not appear to function effectively in clearance of HSV-1. It has been shown that the severity and persistence of HSV-1 lesions do not correlate with serum levels of neutralizing antibodies in infected individuals [6], [7]. HSV-1 encodes type 1 transmembrane glycoproteins, glycoprotein E (gE) and glycoprotein I (gI), that are displayed on the surface of infected cells and virions. Together they function as a receptor for the Fc region of human immunoglobulin G (IgG) [8], [9] and have also been implicated in cell-to-cell spread of virus [10], [11]. In addition, gE is required for HSV-1 movement inside both neuronal and epithelial cells [12]–[15]. The Fc receptor function of gE-gI, which hinders access to the IgG Fc region and thus allows HSV-infected cells to escape recognition by Fc-dependent effector cells, may serve as a mechanism to block antibody-related host defenses [16].

The gE-gI heterodimer is found on the surface of both virions and infected cells [8], [17]. It has been proposed that endocytosis signals in the cytoplasmic tails of HSV and/or VZV gE and gI [18]–[20] result in uptake of gE-gI into intracellular compartments of infected cells via clathrin-mediated endocytosis [21]–[23]. At neutral pH and the slightly basic pH of the cell surface, the gE-gI heterodimer displays a strong binding affinity (K_D_∼340 nM) for the Fc regions of human IgG1, 2, and 4 [8], [24]. gE alone binds to human Fc with an affinity ∼100-fold weaker than the gE-gI heterodimer (K_D_∼30 µM) [25], whereas gI alone shows no Fc or IgG binding activity [26]. Although endocytosis of gE-gI has been confirmed [22], [23], gE-gI-mediated uptake of IgG bound to antigen into intracellular compartments and the fate of potentially endocytosed IgG had not been investigated. However, the binding affinity of gE-gI for IgG was shown to be pH dependent, with the heterodimer displaying strong binding activity at pH 7.4 and no binding below pH 6.0 [25]. This suggested that any IgG that was endocytosed along with gE-gI would dissociate from gE-gI at the acidic pH of endosomes and degradative intracellular compartments, providing a potential mechanism for HSV-1 to facilitate degradation of anti-viral IgGs.

**Figure 1:**
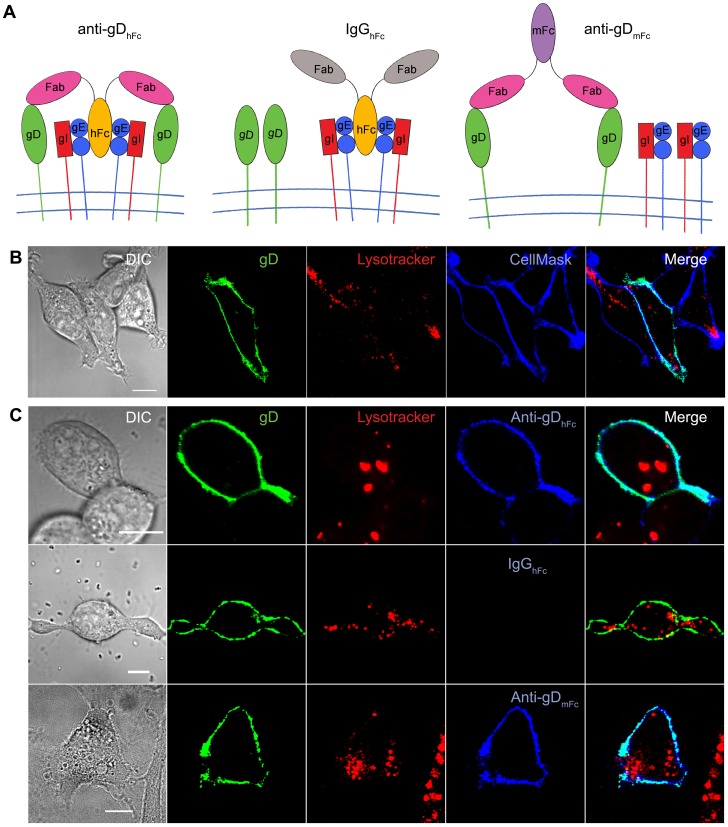
Cell surface complexes and analysis of HSV-1 gD localization. (A) Schematic diagrams of ABB and non-ABB complexes. Left: ABB complex containing HSV-1 gE-gI, anti-gD_hFc_, and gD (gE-Fc interaction as suggested by the crystal structure of a HSV-1 gE-gI/Fc complex [77]); middle: IgG_hFc_ bound to gE-gI, but not gD; right: anti-gD_mFc_ bound to gD but not gE-gI. (B,C) Representative confocal slices (from three independent experiments in which ≥30 cells were analyzed) showing localization of gD-Dendra2 expressed in HeLa cells with Lysotracker (red) and CellMask (blue) (panel B) or Lysotracker (red) and a labeled IgG (blue) (panel C). Regions of green-blue co-localization appear cyan. Scale bar = 10 µm.

Antibodies specific for HSV-1 antigens can be simultaneously bound at the surface of HSV-infected cells to gE-gI via their Fc region and to a cell surface antigen by their antigen-binding fragments (Fabs) (Figure 1A) [27]. This process, which is known as antibody bipolar bridging (ABB), may be a strategy to prevent the host from utilizing anti-HSV-1 antibodies in immune responses. It has been shown that gE-gI can mediate ABB on the surface of HSV-infected cells [27] and that gE-gI is required to prevent host immune functions that require Fc binding, such as complement activation and antibody-dependent cell-mediated cytotoxicity, under conditions in which bipolar bridging could occur [28]–[30].

Although the participation of gE-gI in ABB has been demonstrated [27], questions regarding the fate of a cell surface ABB complex remain. Given that the cytoplasmic tails of gE and gI include YXXφ and dileucine motifs that trigger endocytosis [18], [31], [32], a bound IgG and its associated antigen might be endocytosed together with gE-gI. In this scenario, a cell surface viral antigen that is not normally endocytosed could be transported to acidic compartments not in its trafficking itinerary. The IgG/antigen complex would then presumably enter a degradative pathway after dissociating from gE-gI at acidic pH. Alternatively, the gE-gI/IgG/antigen complex could dissociate prior to endocytosis, so that either the IgG/antigen complex remained at the cell surface while gE-gI was endocytosed, or the antigen remained at the cell surface while the gE-gI/IgG complex was endocytosed. A final possibility is that formation of an ABB complex would inhibit gE-gI endocytosis so that the entire complex remained at the cell surface.

Here we describe an *in vitro* system using transiently-transfected human cells to monitor the internalization and trafficking of gE-gI, IgG, and an antigen under conditions in which ABB could or could not occur. Under conditions that favored ABB between an antigen and gE-gI, we found that the antigen and its bound antibody were internalized and targeted to compartments with lysosomal characteristics. By contrast, only background levels of IgG and antigen were found in intracellular compartments when the conditions did not permit IgG binding to gE-gI and/or ABB complex formation. We also demonstrated that the internalized surface antigen, HSV-1 gD, was localized in lysosomal compartments, presumably for degradation. These results suggest that ABB promotes the uptake of IgG and associated viral antigens, which are directed to intracellular degradative compartments that are not part of their normal trafficking itineraries. This provides a mechanism for HSV-infected cells to evade IgG-mediated immune responses by clearing host IgG and viral antigens from the cell surface.

## Results

### Components of a system to investigate ABB complexes

To create a model system to study the internalization and trafficking of ABB complexes, we co-expressed HSV-1 gE-gI and a cell surface viral antigen, HSV-1 gD, in a human cell line (HeLa) and then determined the effects of adding IgGs that either could or could not form bridged complexes (Figure 1A). We chose to investigate ABB trafficking in transfected cells rather than HSV-1–infected cells because viral proteins other than gE, gI and gD could introduce factors that might confound or obscure gE-gI–mediated effects. For example, HSV-1 gM can reroute gD from the cell surface to the trans-Golgi network [33], [34].

We first established conditions under which we could express gE-gI and characterize its interactions with different IgGs. To avoid potential toxicity of herpes virus glycoproteins in stable cell lines [35]–[37], we expressed the proteins transiently in mammalian cells. To ensure equal levels of expression of gE and gI in the same cell, we created a bicistronic gE-gI construct (Figure S1A) using F2A, a picornavirus 2A-like peptide sequence [38]–[40]. We used 3-D confocal immunofluorescence imaging to analyze gE and gI expression from the bicistronic construct in fixed HeLa cells. Cells expressing gE and gI exhibited intracellular staining for both proteins (Figure S1B), similar to the reported localization pattern of VZV gE [41]. Cells transfected with the gE-F2A-gI construct showed comparable levels of both proteins, and all cells that expressed one of the proteins also expressed the other, thus the 2A-containing construct successfully enabled expression of both gE and gI. Therefore staining with anti-gE could be used to verify expression of both gE and gI in the transfected cells.

The next component of our model system involved expression of a membrane-bound antigen. We chose HSV-1 gD, a cell surface glycoprotein found on HSV-1 virions and infected cells [42] for which fusion of the cytoplasmic tail to a fluorescent protein did not affect function in viruses [43]. gD binds to host receptors on target cells, and together with other HSV-1 glycoproteins including gB, gH and gL, it is required for HSV-1 infection in cultured cells [44]. The cytoplasmic tail of gD does not include known endocytosis motifs [45], thus gD would be expected to be primarily localized to the cell surface. We fused the cytoplasmic tail of gD to a fluorescent protein (Dendra2 or Cerulean) so that the localization of gD could be directly tracked (gD-Dendra2) or indirectly determined using an anti-GFP antibody (gD-Cerulean) that would not interfere with the binding of antibodies to the gD ectodomain. We created a plasmid to express gD alone and also a tricistronic construct to express gD together with gE and gI by including a second picornavirus 2A-like peptide sequence. The tricistronic construct directed expression of gE, gI and gD (Figure S1A–C), which could also be achieved by co-transfection of the gE-gI bicistronic construct together with the gD expression vector. Confocal immunofluorescence imaging of cells transfected with the gD expression plasmid alone, the gE-gI bicistronic and gD plasmids, or the tricistronic plasmid showed gD primarily at the cell surface, where it colocalized with a plasma membrane marker (CellMask), and only background staining in the cytosol and intracellular organelles (Figure 1B,C; Figure S1D).

The final component of our model system was a set of antibodies that could bind to both gE-gI and gD, only to gD, or only to gE-gI. HSV-1 gE-gI exhibits a species preference for IgGs by binding human IgG (hIgG), but not mouse IgG (mIgG) [30], [46]. Thus for the anti-gD antibodies, we constructed two forms of the monoclonal anti-gD antibody HSV8 [47], [48]: one in which the Fabs were fused to a hIgG1 Fc (anti-gD_hFc_), which could bind to gE-gI via its Fc and to gD via its Fabs, and one in which the Fabs were fused to a mIgG2a Fc (anti-gD_mFc_), which could bind to gD but not to gE-gI (Figure 1A). For the antibody that could bind to gE-gI but not gD (IgG_hFc_), we used a hIgG against an irrelevant antigen (HIV-1 gp120). To verify that the antibodies exhibited the expected binding properties, we used confocal fluorescence microscopy to demonstrate that anti-gD_hFc_ bound to transfected cells expressing only gD or only gE-gI, that IgG_hFc_ bound to cells expressing gE-gI, but not to cells expressing only gD, and that anti-gD_mFc_ bound to cells expressing gD but not to cells expressing only gE-gI (Figure 1C; Figure S2). None of the antibodies bound to cells expressing only Dendra2 or to non-transfected cells in the same field of view (Figures S1E, 2).

### Characterization of gE-gI- and gD-expressing cells

Cells expressing the gE-gI heterodimer specifically internalized hIgG but not mIgG (Figure S2), correlating with the binding properties of gE-gI [24]. In contrast, gE-gI–negative cells in the same field, cells transfected with gI alone, and untransfected cells did not internalize detectable amounts of IgG under identical assay and imaging conditions (data not shown). Internalization of hIgG in gE-gI–expressing cells was observed when hIgG was incubated at pH 7.4 but not at pH 6.0 (Figure S2), correlating with the pH-dependent binding interaction between gE-gI and IgG [25]. Staining for gE and internalized hIgG showed some colocalization, although many intracellular compartments were positive for either gE alone or hIgG alone, consistent with dissociation of hIgG from gE-gI after internalization (Figure S2,3). In order to determine if antibody binding to gD could trigger IgG and/or gD internalization via a gE-gI–independent mechanism, we repeated the internalization experiments using cells expressing only gD that had been incubated with anti-gD_hFc_, anti-gD_mFc_ or IgG_hFc_. Bound anti-gD antibodies remained on the cell surface with little or no internalization and the distribution of gD-Dendra2 was not significantly changed by incubation with anti-gD_hFc_, anti-gD_mFc_ or IgG_hFc_ (Figure 1C). These results verified that gD localization was unaffected by incubation with antibodies in the absence of gE-gI.

### Internalization of ABB complexes

**Figure 2:**
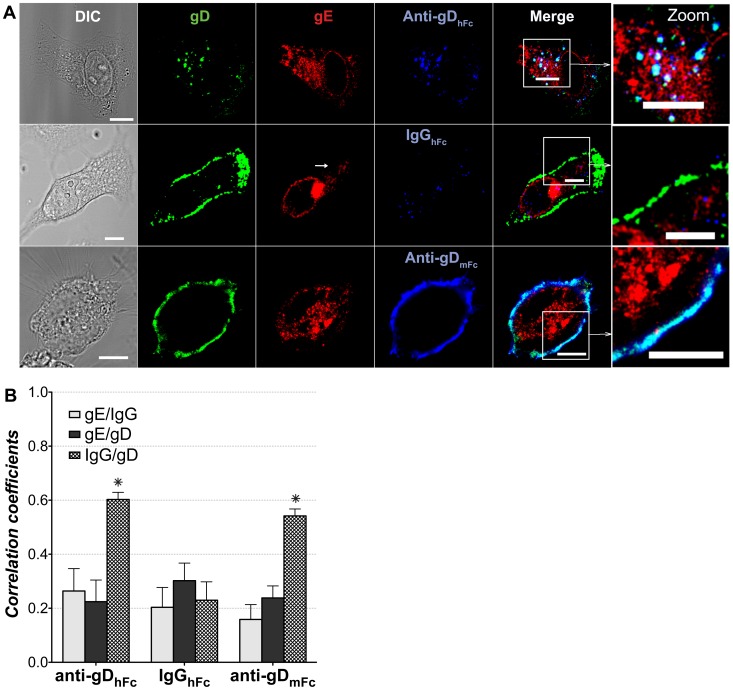
Localization of gE-gI, gD and IgG under ABB-permissive and non-permissive conditions. HeLa cells transiently expressing gE-gI and gD-Dendra2 were incubated for 60 min at 37°C and 5% CO_2_ with labeled IgGs (blue) and then fixed and processed for immunofluorescence using antibodies against gE (red) and gD-Cerulean (green). (A) Representative confocal slices (from three independent experiments in which ≥30 cells were analyzed) from cells treated with anti-gD_hFc_ (top panel), IgG_hFc_ (middle panel), or anti-gD_mFc_ (bottom panel). Regions of gE-gD colocalization appear yellow; regions of gD-IgG colocalization appear cyan, regions of gE-IgG colocalization appear magenta, and regions of triple colocalization appear white. Scale bar = 10 µm. (B) 3-D thresholded Pearson correlation coefficient analyses for data from ≥30 cells. Correlation coefficients are presented as the mean and standard deviation from experiments repeated at least three times. Asterisks (*) indicate a significant difference of colocalization compared to other members in the same category (p value<0.01).

We next conducted internalization experiments on cells expressing gE-gI and gD under conditions in which ABB complexes either could or could not form. HeLa cells were transiently transfected with the gE-F2A-gI and gD-Cerulean vectors and then incubated with fluorescent-labeled anti-gD_hFc_, anti-gD_mFc_, or IgG_hFc_. Cells were fixed and stained with antibodies against gE and GFP (to localize gD-Cerulean). In samples treated with anti-gD_hFc_, gD was no longer found at the plasma membrane, but in intracellular compartments, whereas in samples treated with either IgG_hFc_ or anti-gD_mFc_, gD was localized to the cell surface, with little or no staining in intracellular compartments (Figure 2A). Similar results were found when cells were treated with unlabeled versions of the three antibodies (Figure S3A). These results suggested that under conditions in which an antibody (anti-gD_hFc_) can bind to both gD and gE-gI, gD was internalized by gE as a complex with the antibody, whereas when the antibody could bind only to gD (anti-gD_mFc_) or only to gE-gI (IgG_hFc_), gD remained at the cell surface. As expected, anti-gD_hFc_ and IgG_hFc_ were internalized, whereas anti-gD_mFc_ remained at the cell surface where it colocalized with gD (Figure 2A). When gD was internalized by addition of the anti-gD_hFc_ antibody, gD and anti-gD_hFc_ localized to the same compartments, suggesting they remained as a complex, whereas gE did not colocalize with either anti-gD_hFc_ or with gD in intracellular compartments, consistent with dissociation of the antibody-gD complex from gE-gI in acidic compartments. These observations were confirmed by quantitative 3-D colocalization analyses, which demonstrated significant colocalization of anti-gD_hFc_ with gD (resulting from colocalization in intracellular compartments) but no colocalization above background for gE with either IgG or with gD (Figure 2B). As expected, we observed significant colocalization of anti-gD_mFc_ with gD, which we attributed to their binding interaction at the cell surface, but no significant colocalization of IgG_hFc_ with gD, consistent with its inability to bind gD. The lack of colocalization of IgG_hFc_ with gE again suggested pH-dependent dissociation of this gE-gI ligand after internalization into acidic intracellular compartments.

Results in fixed cells were confirmed by live cell imaging. Cells expressing gE-gI and gD were pulsed with labeled anti-gD_hFc_, anti-gD_mFc_ or IgG_hFc_, incubated in the absence of antibody, and then stained with CellMask, a plasma membrane marker. Figure S3B shows confocal images obtained 65 minutes after the labeled antibodies were added. As demonstrated by colocalization with CellMask staining at the plasma membrane, gD remained at the cell surface when cells were incubated with anti-gD_mFc_ or IgG_hFc_, but was internalized when cells were incubated with anti-gD_hFc_.

### Trafficking of ABB complexes to lysosomes

**Figure 3:**
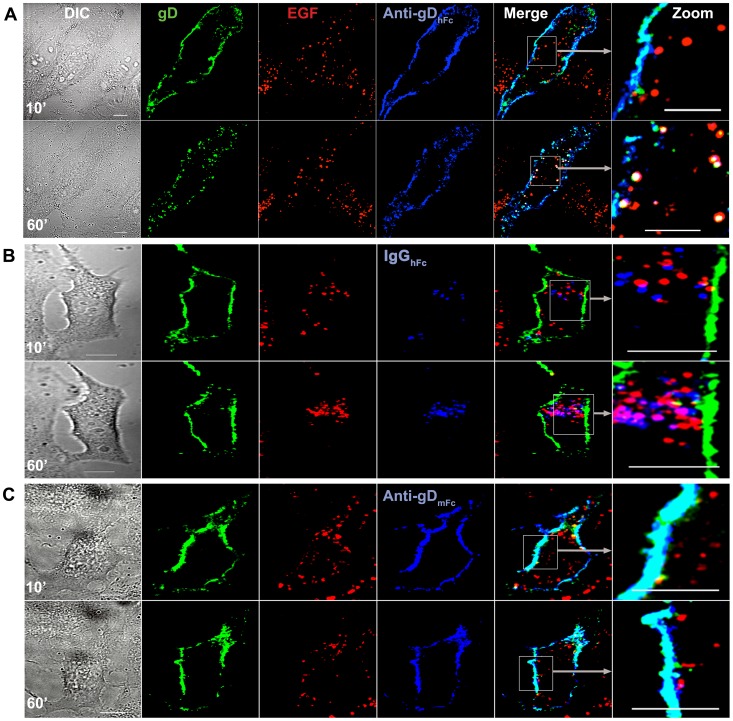
Lysosomal trafficking of HSV-1 gD and IgG under ABB-permissive and non-permissive conditions. Representative confocal slices from early (10 min) and late (60 min) time points from live cell imaging of HeLa cells expressing gE-gI and gD-Dendra2 (green) incubated with EGF (red) and either anti-gD_hFc_ (A), IgG_hFc_ (B) or anti-gD_mFc_ (C) (blue). Regions of EGF-gD colocalization appear yellow; regions of gD-IgG colocalization appear cyan, regions of EGF-IgG colocalization appear magenta, and regions of triple colocalization appear white. Three independent experiments were performed, each with analysis of ≥5 cells. Scale bar = 10 µm.

We next used live cell imaging to follow the intracellular trafficking of ABB complex components and a lysosomal marker, epidermal growth factor (EGF). EGF binds its cell surface receptor to form a complex, which is internalized and traverses the low pH environment of early endosomes, multi-vesicular bodies/late endosomes, and is then degraded within lysosomes [49]–[52]. HeLa cells transiently expressing gE, gI, and gD–Dendra2 were co-incubated with fluorescent-labeled EGF and a labeled version of either anti-gD_hFc_, IgG_hFc_ or anti-gD_mFc_ and 4-D live confocal imaging was performed. At early time points in samples incubated with anti-gD_hFc_, gD and anti-gD_hFc_ fluorescence was localized at the cell surface, and the small amount of gD and anti-gD_hFc_ fluorescence observed intracellularly was not in EGF-positive compartments (Figure 3A; 10 min panel). At later time points, increasing numbers of triple-positive intracellular vesicles staining for EGF, gD and anti-gD_hFc_ were observed (Figure 3A; 60 min panel; Movie S1). These results are consistent with a model for ABB in which anti-gD_hFc_–gD complexes internalized by gE-gI into endosomal vesicles dissociated from gE-gI at low pH and then trafficked into EGF-positive lysosomes. By contrast to gD trafficking under ABB conditions, gD fluorescence remained predominantly at the cell surface at all time points in cells treated with either IgG_hFc_ or anti-gD_mFc_ (Figure 3B,C; Movies S2,3). IgG_hFc_ fluorescence was largely intracellular in EGF-negative compartments at early time points (10 min) and in EGF-positive compartments at later time points (60 min) (Figure 3B; Movie S2), consistent with internalization of IgG_hFc_ bound to gE-gI and subsequent dissociation and targeting to EGF-positive lysosomal compartments. Anti-gD_mFc_ fluorescence remained mainly at the cell surface throughout the experiment (Figure 3C; Movie S3), consistent with binding to cell surface gD without internalization.

**Figure 4:**
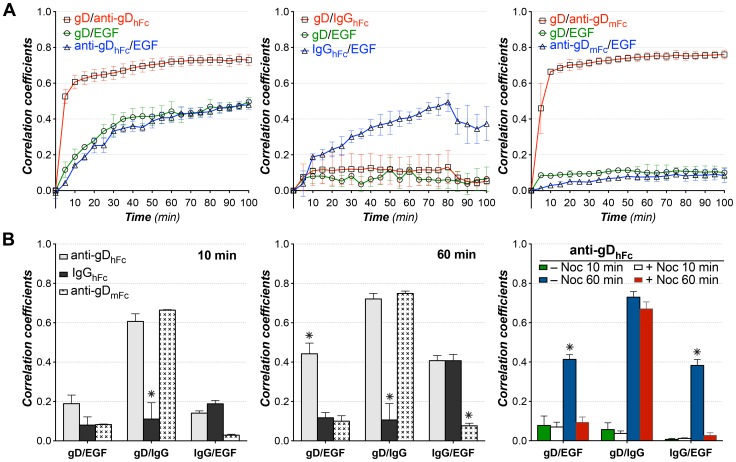
Pairwise colocalization analysis for ABB and non-ABB complexes with EGF. 3-D thresholded Pearson correlation coefficient analyses (presented as the mean and standard deviation) as a function of time for data from ≥5 live cells in three independent experiments for each experimental condition. (A) HeLa cells expressing gE-gI and gD-Dendra2 were incubated with labeled EGF and either anti-gD_hFc_ (left), IgG_hFc_ (middle) or anti-gD_mFc_ (right). Correlation coefficients are shown for gD versus IgG (red curve, open squares), gD versus EGF (green curve, open circles) and EGF versus IgG (blue curve, open triangles). B) Histograms comparing correlations at 10 min (left) and 60 min (middle) time points. Right panel shows correlations for gD versus IgG, gD versus EGF, and EGF versus IgG in anti-gD_hFc_ samples treated with nocodazole. Asterisks (*) indicate a significant difference of colocalization compared to other members in the same category (p-value<0.05).

Statistical analyses of pairwise 3-D colocalization as a function of time showed significant colocalization of gD with the two anti-gD antibodies, but not with IgG_hFc_, at time points after 10 min of incubation, as expected since only the anti-gD antibodies should be bound to gD throughout the experiment (Figure 4A). When incubated with anti-gD_hFc_, gD became steadily more colocalized with EGF and anti-gD_hFc_ after 10 min, whereas EGF was colocalized with IgG_hFc_, but not gD, at later time points when incubated with IgG_hFc_. When incubated with anti-gD_mFc_, gD did not colocalize with EGF. Addition of the microtubule depolymerizing agent nocodazole [53] to cells incubated with anti-gD_hFc_ eliminated the colocalization of EGF with gD and with anti-gD_hFc_, but did not disrupt binding of anti-gD_hFc_ to gD, as demonstrated by colocalization of gD and anti-gD_hFc_ (Figure 4B). These results demonstrated that cell surface gD was exclusively internalized and targeted into EGF-positive lysosomes under ABB conditions and that this trafficking required intact microtubules.

To confirm these results, the experiments were repeated using live cells incubated with Lysotracker as a lysosomal marker [54]. Statistical analyses of pairwise colocalizations as a function of time showed similar trends (Figure S4A,B): cells incubated with anti-gD_hFc_ showed three pairwise colocalizations (gD with anti-gD_hFc_, gD with Lysotracker, and anti-gD_hFc_ with Lysotracker), cells incubated with IgG_hFc_ showed one colocalization (IgG_hFc_ with Lysotracker), and cells incubated with anti-gD_mFc_ showed one colocalization (gD with anti-gD_mFc_).

### Trafficking of ABB complexes through recycling endosomes

To further investigate intracellular trafficking of the components of the ABB complex, we conducted live cell imaging experiments using labeled transferrin (Tf). Iron-loaded Tf bound to transferrin receptor (TfR) at the slightly basic pH of the cell surface enters the cell through receptor-mediated endocytosis, where it traffics through endosomes, including early and recycling endosomes [55]. The low pH of acidic endosomes triggers release of iron from Tf in a receptor-mediated process [56]–[58]. Iron-depleted Tf complexed with TfR recycles back to the cell surface, where completely iron-free Tf (apo-Tf) dissociates from TfR upon encountering the extracellular pH [55]. Several rounds of endocytosis may be required to fully release iron from Tf, thus partially iron-loaded Tf inside endosomes can be recycled to the cell surface as a complex with TfR, where it would remain associated with TfR, allowing another round of receptor-mediated endocytosis into acidic compartments to facilitate release of remaining iron from Tf [59].

**Figure 5:**
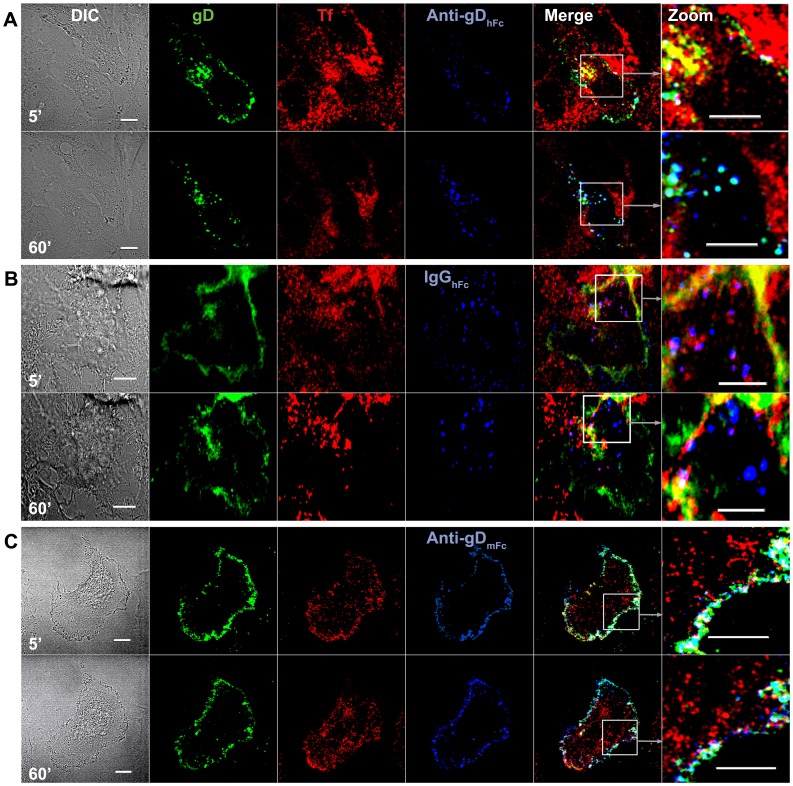
Endosomal trafficking of HSV-1 gD and IgG under ABB-permissive and non-permissive conditions. Representative confocal slices (from three independent experiments, each involving ≥5 cells) from early (5 min) and late (60 min) time points from live cell imaging of HeLa cells expressing gE-gI and gD-Dendra2 (green) incubated with Tf (red) and anti-gD_hFc_ (A), IgG_hFc_ (B) or anti-gD_mFc_ (C) (blue). Regions of Tf-gD colocalization appear yellow, regions of gD-IgG colocalization appear cyan, regions of Tf-IgG colocalization appear magenta, and regions of triple colocalization appear white. Scale bar = 10 µm.

As described for the experiments with EGF, HeLa cells transiently expressing gE, gI, and gD–Dendra2 were co-incubated with fluorescent-labeled iron-loaded Tf and a labeled version of either anti-gD_hFc_, IgG_hFc_ or anti-gD_mFc_, and 4-D live cell confocal imaging was performed. At an early time point (5 min), cells incubated with anti-gD_hFc_ showed anti-gD_hFc_ and gD labeling at the cell surface and inside the cell, with many intracellular gD–anti-gD_hFc_ complexes found in the same compartment as Tf (Figure 5A). At later time points, fewer Tf-positive compartments were also positive for gD and anti-gD_hFc_ (Figure 5A; Movie S4). Taken together with results demonstrating trafficking of ABB complexes to lysosomes (Figure 3,4) and what is known about the Tf-TfR trafficking, these observations suggested that internalized gD–anti-gD_hFc_ complexes first accumulated in Tf-positive early endosomal compartments, but were subsequently sorted into lysosomes after anti-gD_hFc_–gD complexes dissociated from gE-gI, while Tf recycled as a complex with its receptor back to the plasma membrane. IgG_hFc_ also colocalized with Tf at the 5 min time point, but not at later time points (Figure 5B), consistent with gE-gI–mediated internalization of IgG_hFc_ into Tf-positive compartments followed by a diverging intracellular itinerary for IgG_hFc_ and Tf, with IgG_hFc_ being routed to lysosomes after dissociation from gE-gI. After 60 min, some regions at the cell surface showed colocalization between gD and Tf (Figure 5B; Movie S5). As gD was shown to remain at the cell surface upon addition of IgG_hFc_ to gE-gI–expressing cells (Figures 2B,3B,5C), the colocalization of gD with Tf at the cell surface at later time points likely represents colocalization of gD with cell surface TfR complexed with Tf that had not completely released its iron. For similar reasons, gE-gI–expressing cells treated with anti-gD_mFc_ also showed colocalization of gD with Tf (and co-localization of anti-gD_mFc_ with Tf) at the cell surface at later time points (Figure 5C; Movie S6).

**Figure 6:**
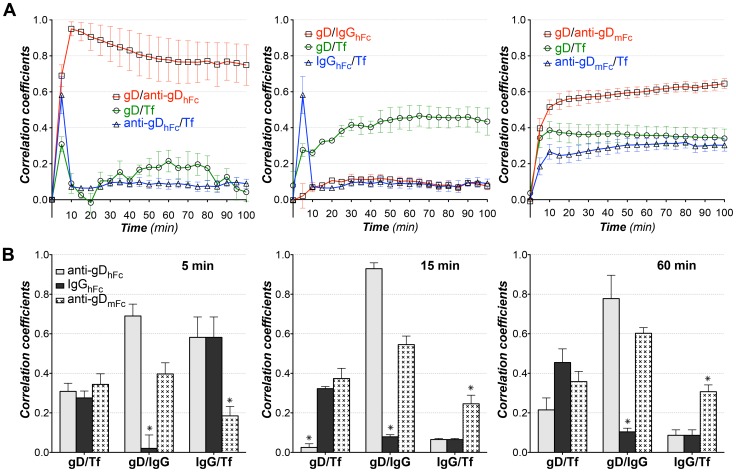
Pairwise colocalization analysis for ABB and non-ABB complexes with Tf. 3-D thresholded Pearson correlation coefficient analyses (presented as the mean and standard deviation) as a function of time for data from ≥5 cells in three independent experiments for each experimental condition. (A) HeLa cells expressing gE-gI and gD-Dendra2 were incubated with Tf and either anti-gD_hFc_ (left), IgG_hFc_ (middle) or anti-gD_mFc_ (right). Correlation coefficients are shown for gD versus IgG (red curve, open squares), gD versus Tf (green curve, open circles) and Tf versus IgG (blue curve, open triangles). (B) Histograms comparing correlations (presented as the mean and standard deviation) at 5 min (left), 15 min (middle) and 60 min (right). Asterisks (*) indicate a significant difference of colocalization compared to other members in the same category (p value<0.05).

Statistical analyses of pairwise 3-D colocalizations showed significant colocalization of gD with the two anti-gD antibodies, but not with IgG_hFc_, at time points after 5 min of incubation, as expected since only the anti-gD antibodies should be bound to gD throughout the experiment (Figure 6). When incubated with anti-gD_hFc_, Tf colocalized at an early time point (5 min) with both gD and anti-gD_hFc_, likely reflecting transient passage of anti-gD_hFc_–gD complexes bound to gE-gI through Tf-positive early endosomes and subsequent re-routing of anti-gD_hFc_–gD complexes through downstream Tf-negative degradative compartments. In cells incubated with IgG_hFc_, the IgG also colocalized at 5 min with Tf, again likely representing endocytosis of IgG_hFc_ into Tf-positive compartments prior to routing to degradative compartments. Consistent with our results and the known Tf trafficking itinerary, colocalization of gD with Tf observed after 5 min in cells incubated with IgG_hFc_ likely reflected colocalization of cell-surface gD with cell-surface Tf receptor-Tf complexes formed after an initial round of Tf endocytosis. This interpretation also explained the observed colocalization of both gD and anti-gD_mFc_ with Tf after the 5 min time point. Tf colocalization with either gD or anti-gD_mFc_ did not significantly change after 5 min (Figure 6). These results supported our hypothesis that in an ABB condition, gD and anti-gD_hFc_ intracellular trafficking followed a lysosomal targeting, but not a rapid endosomal recycling, pathway.

## Discussion

Antibody bipolar bridging (ABB), in which an anti-viral IgG bound to a cell surface antigen also binds to an Fc receptor, has the potential to protect virions and infected cells from IgG-mediated immune responses. gE-gI is an HSV-1 heterodimeric complex that can function as a receptor for human IgGs by binding to their Fc regions [9], thus it can mediate ABB in HSV-1–infected cells. Experiments performed in HSV-1-infected cells to compare the efficacy of IgGs that can or cannot form ABB complexes suggested that bipolar bridging protects HSV-1 and HSV-1–infected cells from antibody- and complement-dependent neutralization [27], antibody-dependent cell-mediated cytotoxicity [30], and granulocyte attachment [29]. Since gE-gI is endocytosed [18], associated IgGs are predicted to be taken into intracellular compartments along with the viral receptor, as we have now demonstrated (Figure S2). The fate of a cell surface ABB complex remained unknown. Given that most antibodies bind protein antigens with high affinities roughly comparable to the nM affinity of gE-gI for IgG [25], it was possible that antigen-antibody complexes would remain associated during gE-gI-mediated endocytosis such that internalization of gE-gI could indirectly cause uptake of cell surface viral antigens, redirecting them and any associated IgGs to acidic intracellular compartments. This would allow the virus to clear the cell surface of viral antigens and anti-HSV-1 IgGs. Here we constructed an *in vitro* system to monitor the trafficking of bipolar bridged complexes involving the HSV-1 Fc receptor gE-gI, the HSV-1 antigen gD, and IgGs.

**Figure 7:**
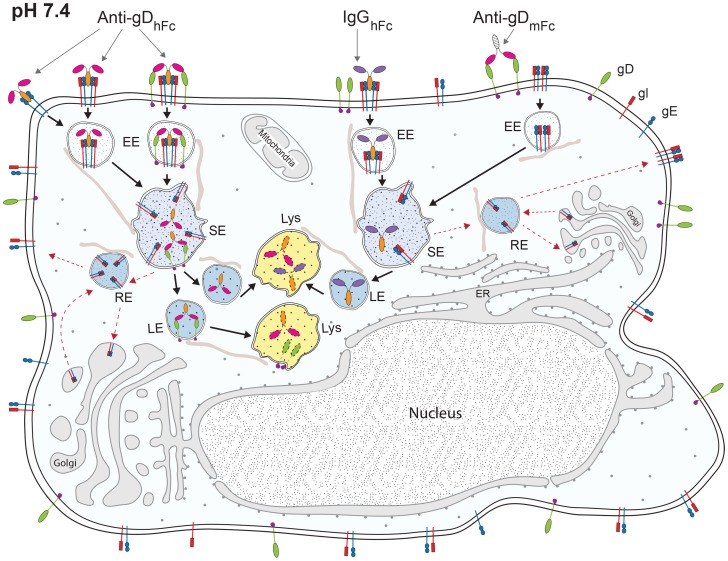
Model of ABB and non-ABB component trafficking. Cell surface ABB complexes (the Fc of anti-gD_hFc_ bound to gE-gI and the Fabs bound to gD) are endocytosed into early endosomes (EE) and sorting endosomes (SE). Upon acidification, the Fc region of anti-gD_hFc_ dissociates from gE-gI but the Fabs remain bound to gD. The IgG-gD complex then enters the lysosomal pathway (Lys) to be degraded. Intracellular trafficking of compartments containing ABB components depends upon intact microtubules (thin straws adjacent to intracellular vesicles). Free gE-gI could be trafficked via a retrograde pathway to the Golgi/ER network and recycling endosomes (RE) before recycling back to the cell surface. IgG_hFc_ bound to gE-gI traffics similarly, but does not recruit cell surface gD into ABB complexes. Anti-gD_mFc_ remains bound to gD at the cell surface.

Cells expressing gE-gI and gD were incubated without IgG or with one of three forms of IgG: anti-gD_hFc_ (binds gD and gE-gI), anti-gD_mFc_ (binds gD only), and IgG_hFc_ (binds gE-gI only) (Figure 1A). We then used confocal fluorescence microscopy to localize the HSV-1 proteins, the IgGs, and intracellular markers. In the absence of IgG, gE-gI was mainly intracellular, whereas gD was primarily at the cell surface (Figure S1C). This distribution did not change appreciably when cells were incubated with IgGs that could not form ABB complexes (anti-gD_mFc_ or IgG_hFc_), but we observed an increase of gD inside intracellular compartments upon addition of anti-gD_hFc_, an IgG that could participate in ABB (Figure 2). In order to objectively evaluate the images, we performed quantitative colocalization studies of ABB components and intracellular markers (Tf for early and sorting endosomes; EGF and Lysotracker for lysosomes) in at least 30 fixed cells and five live cells for each experimental condition. In cells treated with anti-gD_hFc_, these studies showed a significant increase of gD in early endosomes and sorting endosomes at early time points followed by transport of gD into lysosomes, by contrast to the primarily cell surface localization of gD in cells treated with IgG_hFc_ or anti-gD_mFc_ (Figure 3–6). Given that addition of anti-gD_hFc_ or anti-gD_mFc_ or IgG_hFc_ did not cause redistribution of gD to intracellular compartments in cells that did not express gE-gI (Figure 1C), these results demonstrated that ABB complexes were endocytosed along with gE-gI. Although these experiments were done in transfected cells, extrapolating these results to virally-infected cells would provide a mechanism by which HSV-infected cells can sequester both anti-HSV-1 antibodies and viral antigens from the host immune system (Figure 7).

Other studies have demonstrated that ABB resulting from addition of polyclonal anti-HSV-1 antibodies induced patching, capping and extrusion of alpha-herpes viral glycoproteins in some cell lines, another means of removing viral antigens from the surfaces of infected cells [60], [61]. Large cross-linked complexes of gE-gI–IgG–antigen could form upon binding of polyclonal antisera containing anti-gE or anti-gI antibodies. The decision to extrude or endocytose an ABB complex might depend on whether the complex exceeds the ∼200 nm size threshold for clathrin-mediated endocytosis [32].

The sharply pH-dependent binding affinity of the gE-gI–IgG interaction (binding at pH 7.4 but not pH 6.0) [25] suggested that IgGs bound to gE-gI at the slightly basic pH of the cell surface would dissociate upon encountering the acidic pH of endosomes. Since antibody-antigen interactions are normally stable over the same pH range, an antigen-IgG complex would likely remain associated after the IgG dissociated from gE-gI. This assumption is consistent with our data that showed colocalization of gD and anti-gD throughout the course of incubation (Figures 2–6). Additionally, we observed relatively low correlation coefficients for gE colocalization with gD when cells were incubated with anti-gD_hFc_, and for gE with hIgGs when cells were incubated with anti-gD_hFc_ or IgG_hFc_ (Figure 2C). Separation of the intracellular trafficking itineraries of gE-gI and IgG–antigen complexes would allow the bulk of gE-gI molecules to be recycled back to the cell surface, likely passing through the ER-Golgi compartments [62], [63], while IgG–antigen complexes that had dissociated from gE-gI would traffic to increasingly acidic compartments, ultimately being targeted to lysosomes for degradation (Figure 7). This scenario is supported by the preferential colocalization of gD with lysosomal markers when cells were incubated with anti-gD_hFc_ (Figures 3A,4), and IgGs with EGF when cells were incubated with either of anti-gD_hFc_ or IgG_hFc_ (Figures 3A,B,4).

Both degradative and recycling pathways could be used to further the agenda of HSV-1 in infected cells: with each new infective event HSV-1 only needs to evade the host humoral immune system for a finite amount of time before nascent viral particles are competent to spread to uninfected cells and new hosts. Internalization of ABB complexes and consequent degradation or recycling offers an elegant solution for HSV-1 to clear membrane proteins that serve as antigenic targets and to selectively remove anti-HSV-1 antibodies, thereby preventing IgG-mediated immune effector functions.

## Materials and Methods

### DNA constructs for HSV-1 gD, gE and gI expression in mammalian cells

Genes for HSV-1 gE, gI and gD were provided by Dr. Homayon Ghiasi, Cedars-Sinai Medical Research Institute. Single promoter DNA constructs expressing either one or multiple HSV-1 genes were made using the In-Fusion Enzyme kit from Clontech Inc. (Mountain View, CA), which uses a ≥15 bp overlap to fuse DNA fragments and a linearized vector. To prepare the gD constructs, HSV-1 gD and fluorescent protein (Cerulean or Dendra2) gene sequences were amplified by PCR and subcloned into the pCEP4 mammalian expression vector (Life Technologies, Carlsbad, CA) such that the fluorescent protein was fused C-terminal to the cytoplasmic tail of gD (Figure S1A). Confocal imaging using anti-gD antibodies for staining revealed no systematic differences in localizations of gD compared with gD-Cerulean or gD-Dendra2 (data not shown).

To co-express gE and gI from the same mRNA, a spacer encoding a furin protease site (R-X-R/K/X-R) [64], a flexible linker (S-G-S-G) and a picornavirus F2A peptide sequence [65] were inserted between the HSV-1 gE and gI gene sequences in a pCDNA4.0.TO vector [65]–[67] (Figure S1B). The flexible linker between the N-terminal gE protein and the 2A peptide sequence was added to improve the efficiency of the ribosomal skip mechanism [65], [68], [69], and the furin cleavage site was added to remove remnants of the linker and P2A sequences. To construct the gE-gI bicistronic vector, the gE gene was modified by PCR to include a 17 bp overlap and a 5′ Not I site, and the gI gene was modified to insert the furin-linker-F2A sequence at its 5′ (N-terminal) end, and a Kpn I site and a 17 bp overlap at its 3′ (C-terminal) end. Both PCR cassettes were fused onto the pCDNA4.0 vector, previously linearized with NotI and KpnI restriction enzymes, using the In-Fusion enzyme kit (Clontech).

To co-express HSV-1 gE, gI and gD-Dendra2 from a single mRNA, we inserted a second form of 2A sequence (a porcine teschovirus-1-derived 2A (P2A) peptide sequence [70]) between gI and gD-Dendra2 to create a tricistronic construct (Figure S1A). The gE-F2A-gI encoding sequence was modified by PCR to include a 17 bp overlap at the 5′ end of the gE gene and a Kpn I site plus the P2A peptide sequence [70] at the 3′ end of the gI gene. The HSV-1 gD-Dendra2 sequence was modified by PCR to insert the P2A sequence at the 5′ end of the gD gene and an EcoRI site and a 17 bp overlap at the 3′ end of the Dendra2 gene. Both PCR cassettes were fused onto the pCDNA4.0 vector, previously linearized with NotI and EcoRI, using the In-Fusion enzyme kit.

### Expression and purification of antibodies

Genes for the heavy and light chains of the anti-gD antibody HSV8 [48] were provided by Dr. Stephen Mayfield, Scripps Research Institute. The HSV8 light chain gene was modified by PCR to include 5′ and 3′ EcoRI sites and then ligated into the pCDNA3.1 mammalian expression vector (Life Technologies). The portion of the HSV8 heavy chain gene encoding the Fab V_H_ and C_H_1 domains was modified by PCR to include a 5′ XhoI site and a 3′ SpeI site and then subcloned into the pAc-k-Fc baculovirus expression vector (PROGEN Biotechnik), which includes the gene for the human IgG1 Fc (hFc). This gene was modified by PCR to include 5′ and 3′ BamHI sites and then subcloned into the pCDNA3.1 expression vector using the BamHI site. To make an anti-gD antibody with a mouse Fc region (mFc), PCR was used to insert a 5′ BamHI site and a 3′ bridging sequence (21 nucleotides encoding the end of the HSV8 Fab followed by 20 nucleotides encoding the IgG2a mFc). The Fc portion of the mIgG2a gene (InvivoGen, San Diego, CA) was modified by PCR to include the bridging sequence and a 3′ BamHI site. The two products were mixed together and PCR amplified to generate the anti-gD_mFc_ fusion, which was then ligated into the pCDNA3.1 mammalian expression vector (Life Technologies).

Antibodies were expressed by co-transfection of heavy and light chain expression vectors to produce anti-gD_hFc_ and anti-gD_mFc_ (anti-gD antibodies with a human or a mouse Fc region), and IgG_hFc_ (2G12, a human IgG1 antibody against HIV gp120) [71], [72]. The genes were co-expressed by transient expression in 293T cells using a cationic liposome transfection procedure (Lipofectamine 2000, Life Technologies). Six days post-transfection, IgGs were purified from the harvested media by Protein A chromatography using Protein A agarose beads (Pierce/Thermo-Scientific). The IgG_hFc_ antibody was passed over a Superdex 200 16/60 or 10/30 gel filtration column (GE Healthcare) to separate IgG dimer from IgG monomer [73]. The IgG monomer fraction was used for experiments. All of the other IgGs migrated as typical IgG monomers by gel filtration chromatography (data not shown).

### Antibody labeling

Mouse monoclonal anti-gE was from Virusys (Sykesville, MD) and mouse monoclonal anti-gI (2E9) was a gift from Dr. Malini Raghavan (University of Michigan Medical School) [61]. These antibodies and the expressed antibodies (anti-gD_hFc_, anti-gD_mFc_, and IgG_hFc_) were directly conjugated to Alexa fluor (AF) dyes (AF488-NHS, AF568-NHS, or AF647-NHS; Life Technologies, Inc.) according to the manufacturer's protocol. Labeled antibodies were separated from unconjugated dye using a 10,000-kDa cutoff dextran desalting column (Pierce/Thermo-Scientific, Rockford, IL, USA), and the concentration and degree of labeling were determined spectrophotometrically following protocol described by Life Technologies, Inc.

### Cell culture, transfection and preparation

HeLa cells were cultured in Dulbecco's Modified Eagle Medium (DMEM) medium (Life Technologies) supplemented with 10% heat inactivated fetal bovine serum (HyClone, Logan, UT) and 100 µg/mL penicillin/streptomycin mix (Sigma, St. Louis, MO), and incubated at 37°C under a 5% CO_2_ atmosphere. Fully confluent cells were detached using 0.25% (w/v) trypsin-EDTA (Life Technologies) and passaged every 3 days.

Attempts to make stable HeLa cell lines constitutively expressing gE, gI, or both proteins were unsuccessful, presumably due to toxicity associated with expression of these proteins [37]. A stable cell line expressing VZV gE under the control of an inducible promoter was reported [19]. We therefore attempted to use a dual induction system involving expression of gE under the control of a tetracycline-inducible promoter [74] and expression of gI under the control of a cumate-inducible promoter [75]. Although we were able to create a stable HeLa cell line expressing an inducible gE, we were unable to isolate stable cell lines that expressed gI after induction, either in the gE-inducible cell line or in cells that did not express gE (data not shown). We therefore switched to transient transfections to express gE, gI and gD for short periods of time. The gE-F2A-gI, gE-F2A-gI-P2A-gD-Dendra2 and gD-Cerulean expression vectors were transiently transfected either alone or in various combinations into HeLa cells using a cationic liposome transfection procedure (Lipofectamine 2000, Life Technologies). At least 18 hours post-transfection, the cells were treated with 75 µg/mL cycloheximide (Sigma-Aldrich, St Louis, MO) to attenuate intracellular staining of newly synthesized proteins in the endoplasmic reticulum or Golgi apparatus. Two hours later, the cells were rinsed in Hank's Balanced Salt Solution, 10 mM MES pH 6.0, 1% ovalbumin, and 75 µg/mL cycloheximide (M1 buffer) at 37°C for 5 min to remove trace amounts of bovine IgG in the ultra-low IgG serum used for cell culture (the pH was lowered to 6.0 to promote dissociation of any bovine IgG present in the serum that might be bound to cell surface gE-gI). The cells were then incubated at 37°C with 2 µg/mL labeled or unlabeled IgG (either anti-gD_hFc_, anti-gD_mFc_, or IgG_hFc_) in a serum-free Leibovitz's L-15 medium (Life Technologies) and 75 µg/mL cycloheximide at 37°C with 5% CO_2_.

### Cell surface localization of HSV-1 gD

Hela cells transiently expressing only gD-Cerulean or gD-Dendra2 were incubated with 50 nM Lysotracker (Life Technologies), a lysosomal marker conjugated to AF568 dye in L-15 medium (Life Technologies) at 37°C with 5% CO_2_ atmosphere. After 30 min, unincorporated dye was removed by rinsing the preparations three times with fresh, pre-warmed L15 medium. Samples were then stained for 5 min under the same conditions with a plasma membrane marker (0.5 µg/mL CellMask dye; Life Technologies) or a mitochondrial marker (25 nM Mitotracker dye; Life Technologies), which had been labeled with AF647. To analyze the binding specificity and impact of antibodies on the gD-Cerulean or gD-Dendra2 distribution, cells expressing only gD were pulsed for 60 min at 37°C and 5% CO_2_ with either anti-gD_mFc_, anti-gD_hFc_, or IgG_hFc_ conjugated with AF647 dye to a final concentration of 2 µg/mL in L15 medium supplemented with 75 µg/mL of cycloheximide, and then with 50 nM AF568-labeled Lysotracker dye for 5 min under the same conditions. Cells expressing only Dendra2 were included as controls (Figure S1A). Samples were directly imaged live or fixed and processed for immunofluorescence.

### pH-dependent uptake analyses

HeLa cells transiently expressing HSV-1 gE-gI, but not HSV-1 gD, were washed with a M1 buffer (pH 6.0) to remove any serum bovine IgG bound to gE-gI. Cells were then incubated at pH 7.4 or pH 6.0 with AF488-labeled anti-gD_hFc_, anti-gD_mFc_ or IgG_hFc_ (final concentration of 2 µg/mL). After 15 min, cells were rinsed to remove unbound antibodies, and samples were returned to the incubator to allow potential endocytosis of gE-gI–IgG complexes. After 45 min, samples were rinsed three times in sterile 1× PBS and then fixed in 4% paraformaldehyde for 30 min at room temperature (RT) or over-night at 4°C. Fixed cells were processed for immunofluorescence and stained with 5 µg/mL of primary antibodies against HSV-1 gE and gI directly conjugated with AF568 and AF647 dyes, respectively, and with 0.5 µg/mL of the nucleic acid-specific dye 4′,6-diamidino-2 phenylindole dihydrochloride (DAPI) (Life Technologies, Inc.). Samples were imaged and recorded on an Ultra View ERS Rapid Confocal Imager (PerkinElmer, Inc.) using a 100× objective (αPlan-APOCHROMAT 1.46 Oil DIC, Zeiss). AF488, AF546, and AF647 dyes were excited at 488 nm, 568 nm, and 647 nm, respectively, using a multi-line argon/krypton laser (Miles-Griot).

### Characterization of HSV-1 gE-gI and gD localization under ABB-permissive and non-permissive conditions

HeLa cells transiently expressing HSV-1 gE-gI and gD-Dendra2 or gD-Cerulean were incubated for 60 min with AF647-conjugated or with unlabeled anti-gD_hFc_, anti-gD_mFc_, or IgG_hFc_ at a final concentration of 2 µg/mL. Samples fixed in 4% PFA were processed for immunofluorescence and stained with AF568-labeled primary antibodies against gE. Samples incubated with unlabeled anti-gD_hFc_, anti-gD_mFc_, or IgG_hFc_ IgGs were immunostained with 5 µg/mL of anti-gI antibody conjugated with AF647. 3-D multi-channel fluorescence confocal images of both treated and non-treated transfected cells were acquired using a Zeiss Laser Scanning Microscope 510 (LSM510), and analyzed with both Imaris 7.6.1 and Image/Fiji software.

### Fixed cell immunofluorescence confocal microscopy

Transfected cell samples seeded on 12 mm round coverslips or on Lab-Tek chambered coverglass bottom dishes (Thermo Scientific/Nunc, Rochester NY) were pulsed with either labeled or unlabeled anti-gD_hFc_, anti-gD_mFc_ and IgG_hFc_ IgGs. Samples were then fixed in 4% PFA for at least 20 min at RT, rinsed three times in 1× PBS, quenched in 50 mM NH_4_Cl/1× PBS solution for 5 min at RT. Fixed samples were simultaneously blocked and permeabilized in 2% (w/v) chicken serum albumin (CSA)/1× PBS/0.25% (v/v) Triton X-100 for 60 min at RT or 4°C overnight. Incubations with directly-labeled primary antibodies were performed in blocking solution overnight at 4°C. Cells were washed three times in 0.25% (v/v) Triton X-100/1× PBS and 1× PBS solutions before mounting on glass slides using Pro-long GOLD anti-fade mounting media containing DAPI (Life Technologies). Samples were imaged either on the a Zeiss LSM510 Meta NLO with Coherent Chameleon fitted with a 63× objective lens (αPlan-APOCHROMAT 1.45 Oil DIC) or an Ultra View ERS Rapid Confocal Imager (PerkinElmer, Inc.) using a 100× objective (αPlan-APOCHROMAT 1.46 Oil DIC, Zeiss). DAPI was excited at 350 nm/DAPI filter or at 725 nm with a two-photon unit attached to the LSM510 microscope. 3-D confocal stacks were sampled at 0.2–1 µm intervals. Confocal images shown are representative slices from 3-D confocal stacks sampled at 0.5 µm intervals unless otherwise indicated.

### HSV-1 gD and IgG internalization assay

Transfected cells were pulsed for 30 min at 37°C and 5% CO_2_ with AF647-conjugated anti-gD_mFc_, anti-gD_hFc_, or IgG_hFc_ (final concentration 2 µg/mL) in L15 medium supplemented with 75 µg/mL of cycloheximide. Cultures were rinsed with pre-warmed L15 medium to remove excess IgG. After 30 min, AF568-conjugated CellMask (Life Technologies) was added into the medium at a final concentration of 0.5 µg/mL. After 5 min, samples were rinsed with fresh pre-warmed medium, and multi-channel z-stack images were captured using a 63× objective lens (αPlan-APOCHROMAT 1.45 Oil DIC) on a Zeiss LSM510 microscope.

### 4-D confocal microscopy

Transfected cells co-expressing gE-gI and gD-Dendra2 were co-incubated with AF568-conjugated EGF or Lysotracker at a concentration of 50 nM in 75 µg/mL cycloheximide/L15 medium at 37°C and 5% CO_2_. After 30 min, samples in Lab-Tek chambered cover-glass bottom dishes (Thermo Scientific/Nunc) were rinsed to remove excess dye and fresh medium was added. 4-D multi-channel confocal imaging was performed using a 63× objective lens (αPlan-APOCHROMAT 1.45 Oil DIC) on a LSM510 microscope (Zeiss) at a 37°C and a 5% CO_2_ atmosphere using a motorized temperature-controlled stage, an environmental chamber, and a CO_2_ enrichment system. After imaging an initial z-stack (0.5–1 µm section thickness and up to 16 µm total depth), the ZEN 2009 software (Zeiss) was paused and AF647-conjugated anti-gD_mFc_, anti-gD_hFc_, or IgG_hFc_ was added to a final concentration of 2 µg/mL. The software was then re-activated and multi-channel z-stacks were captured approximately every 3 min for at least one hour. Images were acquired and processed by an electron-multiplying CCD (charge-coupled device) camera (Hamamatsu Photonics).

Tf-based experiments were performed similarly except that AF568-conjugated Tf was mixed with AF647-conjugated IgG (concentrations of 1 µg/mL and 2 µg/mL, respectively) before being added into the culture after the 3-D image of the first time point was captured.

### Quantification, statistical analysis and image processing

Imaris 7.6.0, ImageJ, Photoshop CS and Illustrator CS (Adobe) software were used for image processing. Both 3-D and 4-D colocalization analyses were performed using Imaris 7.6.0 software (Bitplane; Zurich, Switzerland), and the thresholded Pearson's correlation coefficient (PCC) in the colocalized volume was determined for each sample. At least 30 transfected cells were examined for each 3-D experiment, and ≥5 live cells were analyzed for each 4-D experiment. Data were based on three or more independent experiments and are presented as mean ± standard deviation. For each pair-wise colocalization analysis, the voxel intensity threshold was set using the background subtraction function as implemented in the Imaris colocalization module [76]. The region of interest dataset was set by a channel based-mask. Correlation values were calculated using the automatic threshold function with a P-value = 1 and a point spread function width of 0.141 µm. Statistical analysis and graphs were done using Prism5 (GraphPad). Two-way ANOVA was performed on each set of mean correlation values and P-values were calculated with a Student-Newman-Keuls post-test.

## Supporting Information

Figure S1 **Characterization of DNA constructs.** (A) Schematics of mammalian expression vectors. CMV indicates a cytomegalovirus promoter, gE, gI and gD are the genes for HSV-1 gE, gI and gD, each including its hydrophobic leader peptide, F2A and P2A indicate sequences resulting in cleavage between the two indicated gene products. (B) Representative image of HeLa cells transiently expressing gE (red) and gI (blue) from the bicistronic gE-gI vector, with nuclei stained in white. (C) Representative image of a HeLa cell transiently expressing gE (red), gI (blue) and gD-Dendra2 (green) from the tricistronic vector. (D) Representative image of a HeLa cell transiently expressing gD-Dendra2 (green), which was stained with 50 nM Lysotracker (red) and 25 nM Mitotracker (blue). (E) Representative image of a HeLa cell transiently expressing cytoplasmic Dendra2, which does not co-localize with Lysotracker or stain with anti-gD IgGs (results are shown for anti-gD_mFc_; similar results were obtained for anti-gD_hFc_ and IgG_hFc_; data not shown). Scale bar = 10 µm.
(TIF)

Figure S2 **pH-dependent binding of gE-gI to human IgG.** Cells transiently expressing gE-gI, but not gD, were pulsed for 60 min at pH 7.4 or pH 6.0 with anti-gD_hFc_ (A), IgG_hFc_ (B) or anti-gD_mFc_ (C) (green). Fixed cells were stained with antibodies against gE (red) and gI (blue). The experiments were repeated at least three times with analysis of ≥30 cells. Scale bar = 10 µm.
(TIF)

Figure S3 **Redistribution of cell surface gD under ABB conditions.** (A) HeLa cells transiently expressing gE-gI and gD-Dendra2 were incubated with unlabeled IgGs (blue) for 60 min and then fixed and processed for immunofluorescence using antibodies against gE (red) and gD-Dendra2 (green). Representative confocal slices from cells treated with anti-gD_hFc_ (top), IgG_hFc_ (middle), or anti-gD_mFc_ (bottom). Regions of gE-gD colocalization appear yellow; regions of gD-gI colocalization appear cyan, regions of gE-gI colocalization appear magenta, and regions of triple colocalization appear white. Scale bar = 10 µm. (B) Live HeLa cells expressing gE-gI and gD-Dendra2 were pulsed with labeled IgGs (blue) for 60 min and then treated with CellMask (red), a plasma membrane marker, for 5 min. Representative confocal slices from cells treated with anti-gD_hFc_ (top), IgG_hFc_ (middle), or anti-gD_mFc_ (bottom). Regions of gE-gD colocalization appear yellow; regions of gD-IgG colocalization appear cyan, regions of gE-IgG colocalization appear magenta, and regions of triple colocalization appear white. The experiments were repeated at least three times with analysis of ≥30 cells. Scale bar = 10 µm.
(TIF)

Figure S4 **Intracellular trafficking and lysosomal targeting of HVS-1 gD and hIgG.** (A) 3-D thresholded Pearson correlation coefficient analyses as a function of time for data from ≥5 live cells in at least three independent experiments for each experimental condition. HeLa cells expressing gE-gI and gD-Dendra2 were incubated with Lysotracker and either anti-gD_hFc_ (left), IgG_hFc_ (middle) or anti-gD_mFc_ (right). Correlation coefficients are shown as the mean and standard deviation for gD versus IgG (red curve, open squares), gD versus Lysotracker (green curve, open circles) and Lysotracker versus IgG (blue curve, open triangles). (B) Histograms comparing correlations at 10 min (left) and 60 min (right) time points. Asterisks (*) indicate a significant difference of colocalization compared to other members in the same category (p value<0.01).
(TIF)

Movie S1 **4-D movie of ABB-dependent trafficking of gD and anti-gD_hFc_ to lysosomes (corresponds to** **Figure 3A** **).** Live cell imaging of HeLa cells expressing gE-gI and gD-Dendra2 (green) incubated with EGF (red) and anti-gD_hFc_ (blue). Regions of EGF-gD colocalization appear yellow; regions of gD-IgG colocalization appear cyan, regions of EGF-IgG colocalization appear magenta, and regions of triple colocalization appear white. 4-D multi-channel confocal imaging was performed using a 63× oil objective lens (αPlan-APOCHROMAT 1.45 Oil DIC) on a LSM510 microscope (Zeiss) and an electron-multiplying charge-coupled device (CCD) camera (Hamamatsu Photonics), controlled by the ZEN 2009 software (Zeiss). Z-stacks (at 1 µm section thickness and up to 16 µm total depth) were captured approximately every 3 min for ∼90 min. The video was recorded at a time resolution of approximately 5 seconds per frame and presented at 10 frames per second. The equatorial planes for z-stack sections are shown on this video.
(AVI)

Movie S2 **4-D movie of trafficking of IgG_hFc_, but not HSV-1 gD, to lysosomes under non-ABB conditions (corresponds to** **Figure 3B** **).** Live cell imaging of HeLa cells expressing gE-gI and gD-Dendra2 (green) incubated with EGF (red) and IgG_hFc_ (blue). Regions of EGF-IgG_hFc_ colocalization appear magenta. 4-D multi-channel confocal imaging was performed under conditions described for Supplementary Movie S1.
(AVI)

Movie S3 **4-D movie showing no trafficking of either gD or anti-gD_mFc_ to lysosomes under non-ABB conditions (corresponds to** **Figure 3C** **).** Live cell imaging of HeLa cells expressing gE-gI and gD-Dendra2 (green) incubated with EGF (red) and anti-gD_mFc_ (blue). Regions of gD-anti-gD_mFc_ colocalization appear cyan. 4-D multi-channel confocal imaging was performed under conditions described for Supplementary Movie S1.
(AVI)

Movie S4 **4-D movie of ABB-dependent trafficking of HSV-1 gD and anti-gD_hFc_ to Tf-negative intracellular compartments (corresponds to** **Figure 5A** **).** 4-D confocal imaging of cells expressing gE-gI and gD-Dendra2 (green) treated with anti-gD_hFc_ (blue) and Tf (red). Regions of Tf-gD colocalization appear yellow; regions of gD-anti-gD_hFc_ colocalization appear cyan, regions of Tf-anti-gD_hFc_ colocalization appear magenta, and regions of triple colocalization appear white. 4-D multi-channel confocal imaging was performed under conditions described for Supplementary Movie S1.
(AVI)

Movie S5 **4-D movie of trafficking of IgG_hFc_, but not HSV-1 gD, to Tf-negative intracellular compartments under non-ABB conditions (corresponds to** **Figure 5B** **).** 4-D confocal imaging of cells expressing gE-gI and gD-Dendra2 (green) treated with IgG_hFc_ (blue) and Tf (red). Regions of Tf-gD colocalization appear yellow; regions of gD-IgG_hFc_ colocalization appear cyan, and regions of Tf-IgG_hFc_ colocalization appear magenta. 4-D multi-channel confocal imaging was performed under conditions described for Supplementary Movie S1.
(AVI)

Movie S6 **4-D movie showing no trafficking of either gD or anti-gD_mFc_ to Tf-negative intracellular compartments under non-ABB conditions (corresponds to** **Figure 5C** **).** 4-D confocal imaging of cells expressing gE-gI and gD-Dendra2 (green) treated with anti-gD_mFc_ (blue) and Tf (red). Regions of Tf-gD colocalization appear yellow; regions of gD-anti-gD_mFc_ colocalization appear cyan, regions of Tf-anti-gD_mFc_ colocalization appear magenta, and regions of triple colocalization appear white. 4-D multi-channel confocal imaging was performed under conditions described for Supplementary Movie S1.
(AVI)
