# Supplementary figures and images for: The Herpes Virus Fc Receptor gE-gI Mediates Antibody Bipolar Bridging to Clear Viral Antigens from the Cell Surface
Source: PLoS Pathog. 2014 Mar 6;10(3):e1003961. doi: 10.1371/journal.ppat.1003961 (PMC3946383; doi:10.1371/journal.ppat.1003961)

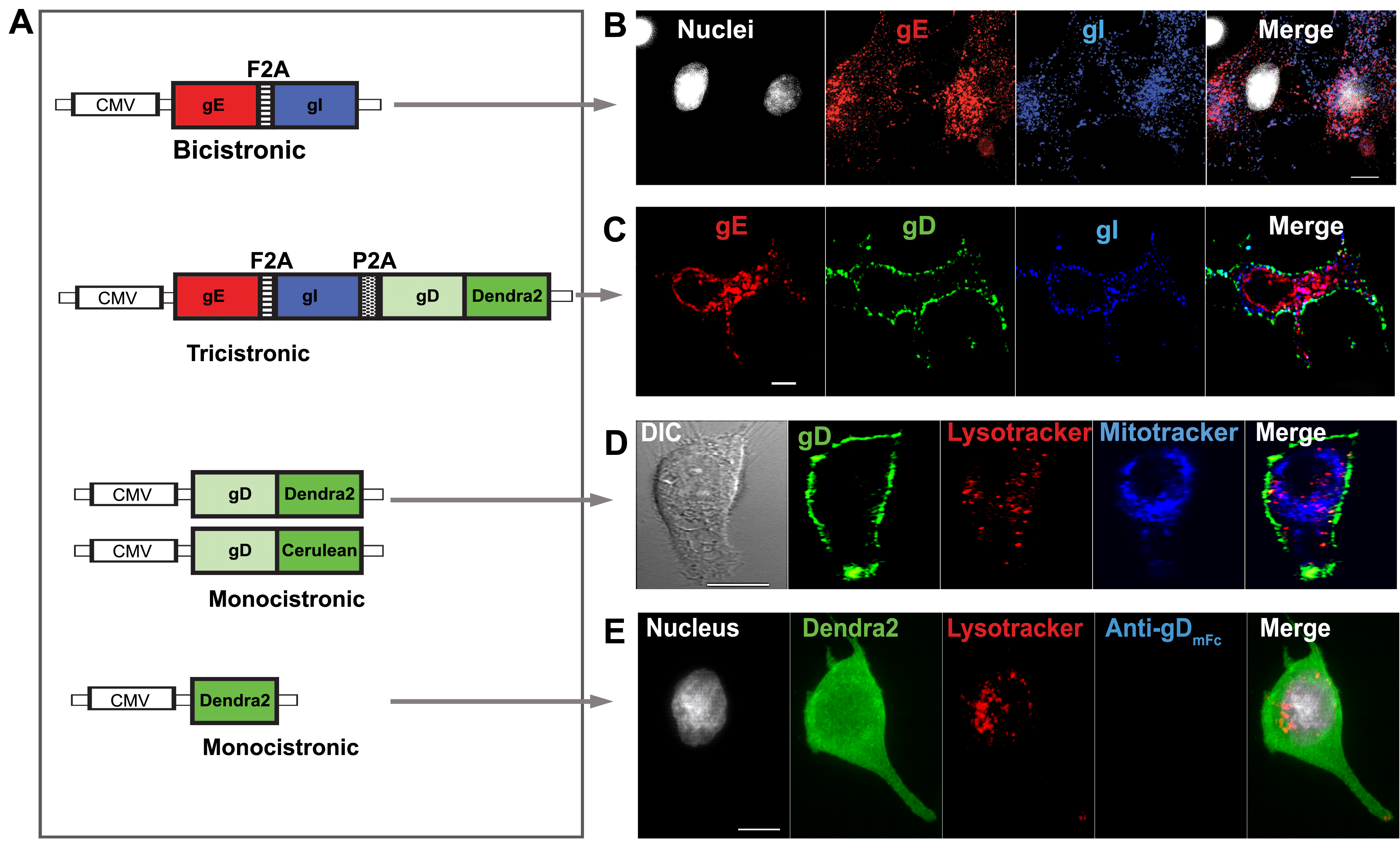

Supplement: Figure S1 — Characterization of DNA constructs. (A) Schematics of mammalian expression vectors. CMV indicates a cytomegalovirus promoter, gE, gI and gD are the genes for HSV-1 gE, gI and gD, each including its hydrophobic leader peptide, F2A and P2A indicate sequences resulting in cleavage between the two indicated gene products. (B) Representative image of HeLa cells transiently expressing gE (red) and gI (blue) from the bicistronic gE-gI vector, with nuclei stained in white. (C) Representative image of a HeLa cell transiently expressing gE (red), gI (blue) and gD-Dendra2 (green) from the tricistronic vector. (D) Representative image of a HeLa cell transiently expressing gD-Dendra2 (green), which was stained with 50 nM Lysotracker (red) and 25 nM Mitotracker (blue). (E) Representative image of a HeLa cell transiently expressing cytoplasmic Dendra2, which does not co-localize with Lysotracker or stain with anti-gD IgGs (results are shown for anti-gDmFc; similar results were obtained for anti-gDhFc and IgGhFc; data not shown). Scale bar = 10 µm. (TIF) [file ppat.1003961.s001.tif]

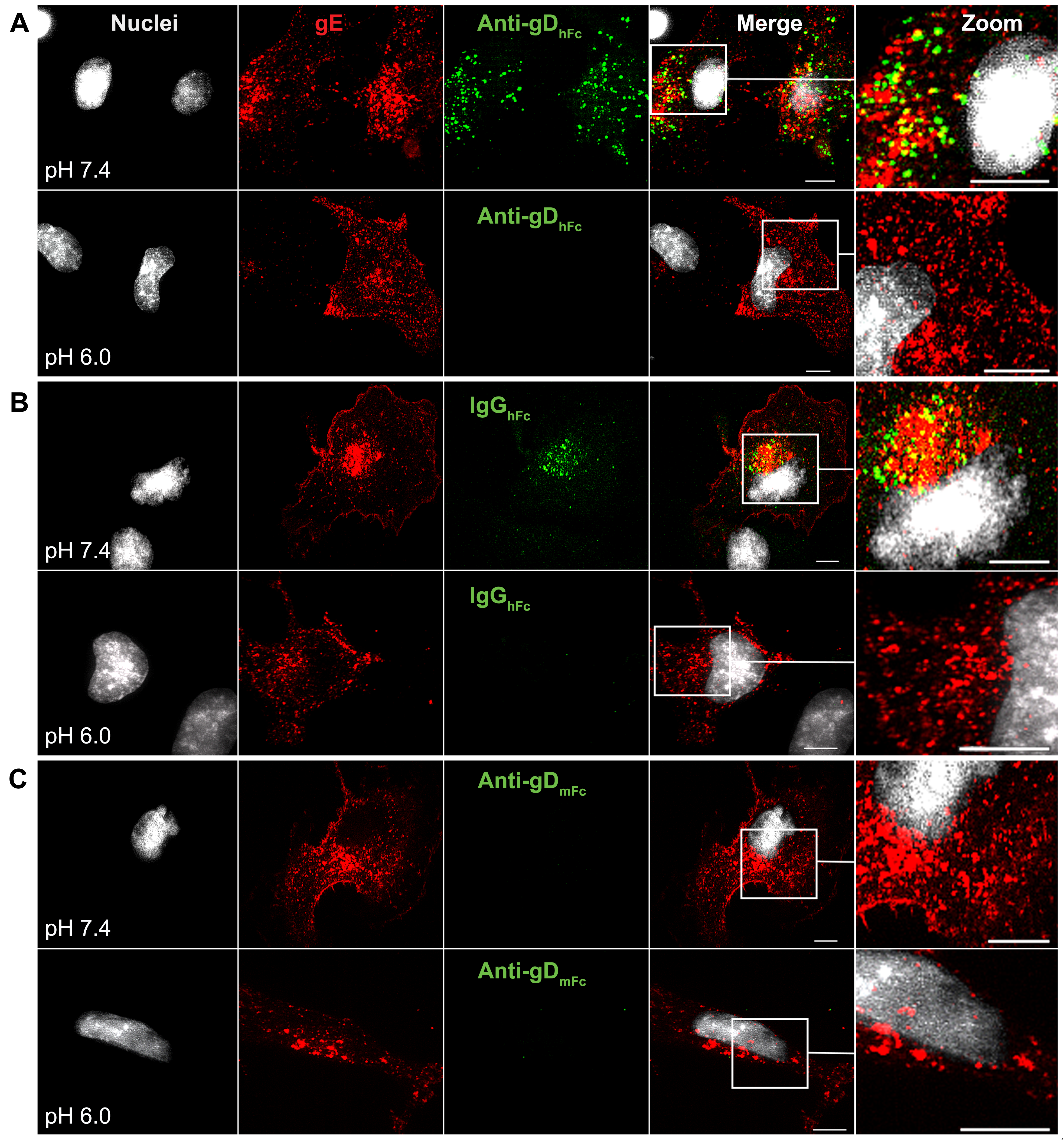

Supplement: Figure S2 — pH-dependent binding of gE-gI to human IgG. Cells transiently expressing gE-gI, but not gD, were pulsed for 60 min at pH 7.4 or pH 6.0 with anti-gDhFc (A), IgGhFc (B) or anti-gDmFc (C) (green). Fixed cells were stained with antibodies against gE (red) and gI (blue). The experiments were repeated at least three times with analysis of ≥30 cells. Scale bar = 10 µm. (TIF) [file ppat.1003961.s002.tif]

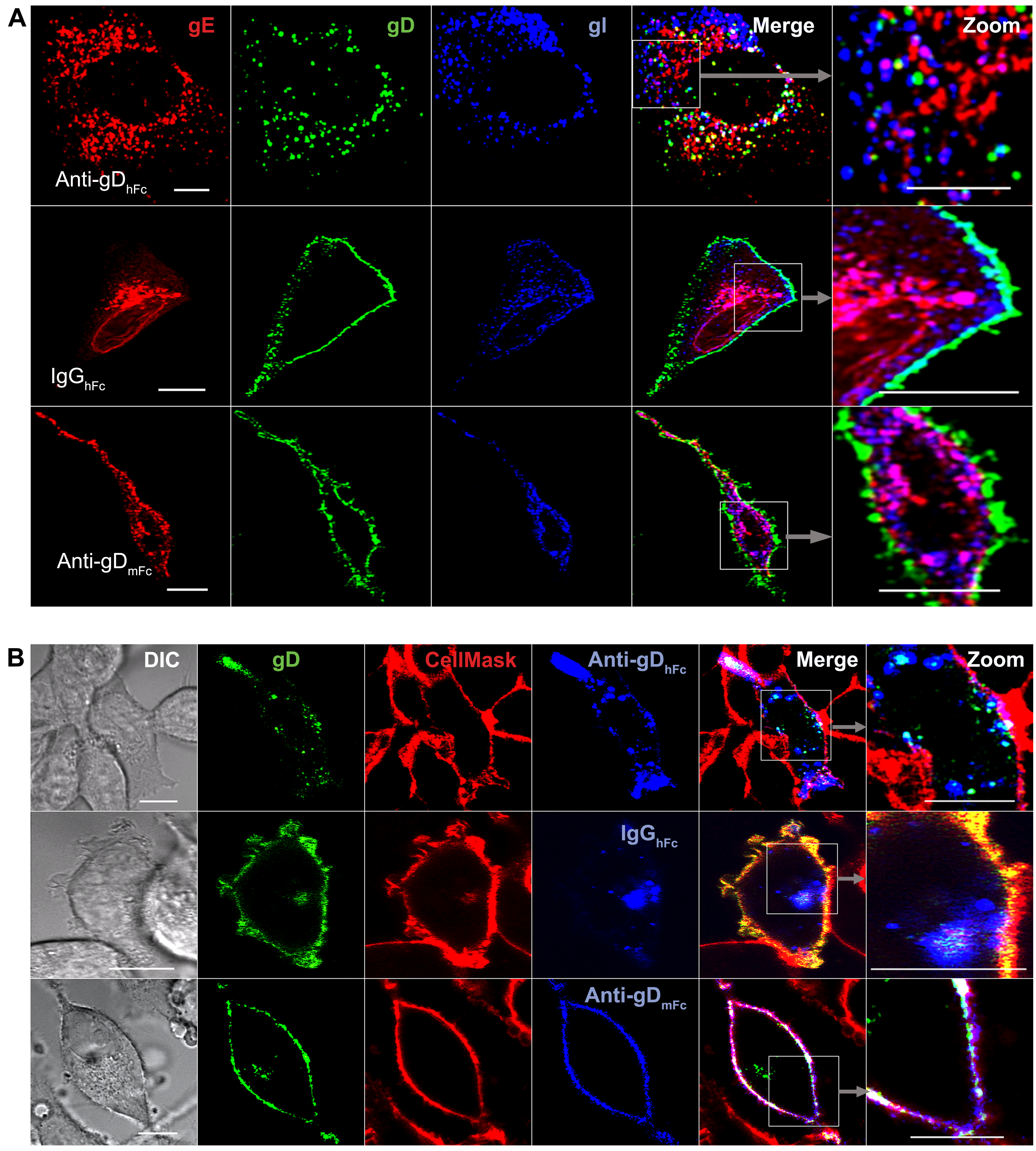

Supplement: Figure S3 — Redistribution of cell surface gD under ABB conditions. (A) HeLa cells transiently expressing gE-gI and gD-Dendra2 were incubated with unlabeled IgGs (blue) for 60 min and then fixed and processed for immunofluorescence using antibodies against gE (red) and gD-Dendra2 (green). Representative confocal slices from cells treated with anti-gDhFc (top), IgGhFc (middle), or anti-gDmFc (bottom). Regions of gE-gD colocalization appear yellow; regions of gD-gI colocalization appear cyan, regions of gE-gI colocalization appear magenta, and regions of triple colocalization appear white. Scale bar = 10 µm. (B) Live HeLa cells expressing gE-gI and gD-Dendra2 were pulsed with labeled IgGs (blue) for 60 min and then treated with CellMask (red), a plasma membrane marker, for 5 min. Representative confocal slices from cells treated with anti-gDhFc (top), IgGhFc (middle), or anti-gDmFc (bottom). Regions of gE-gD colocalization appear yellow; regions of gD-IgG colocalization appear cyan, regions of gE-IgG colocalization appear magenta, and regions of triple colocalization appear white. The experiments were repeated at least three times with analysis of ≥30 cells. Scale bar = 10 µm. (TIF) [file ppat.1003961.s003.tif]

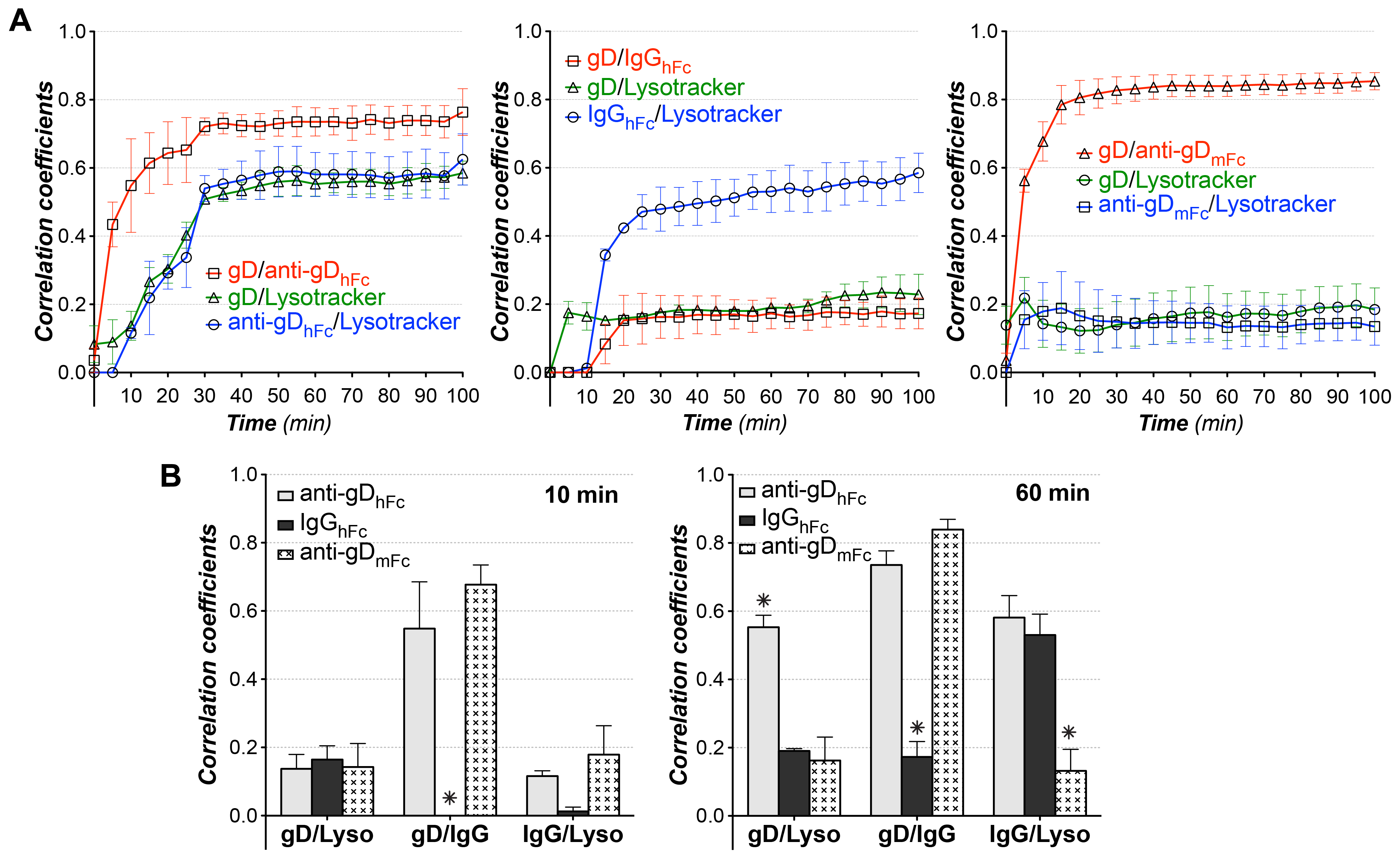

Supplement: Figure S4 — Intracellular trafficking and lysosomal targeting of HVS-1 gD and hIgG. (A) 3-D thresholded Pearson correlation coefficient analyses as a function of time for data from ≥5 live cells in at least three independent experiments for each experimental condition. HeLa cells expressing gE-gI and gD-Dendra2 were incubated with Lysotracker and either anti-gDhFc (left), IgGhFc (middle) or anti-gDmFc (right). Correlation coefficients are shown as the mean and standard deviation for gD versus IgG (red curve, open squares), gD versus Lysotracker (green curve, open circles) and Lysotracker versus IgG (blue curve, open triangles). (B) Histograms comparing correlations at 10 min (left) and 60 min (right) time points. Asterisks (*) indicate a significant difference of colocalization compared to other members in the same category (p value<0.01). (TIF) [file ppat.1003961.s004.tif]
